# Effect of different cocoon stifling methods on the properties of silk fibroin biomaterials

**DOI:** 10.1038/s41598-019-43134-5

**Published:** 2019-04-30

**Authors:** Salvador D. Aznar-Cervantes, Ana Pagan, Beatriz Monteagudo Santesteban, José L. Cenis

**Affiliations:** Instituto Murciano de Investigación y Desarrollo Agrario y Alimentario (IMIDA), Department of Biotechnology. La Alberca (Murcia), E-30150 Murcia, Spain

**Keywords:** Bioinspired materials, Biomedical engineering, Biomaterials - proteins, Biomaterials - cells

## Abstract

Stifling treatments are applied to silk cocoons in order to kill the pupae, preventing the emergence of moths and allowing to preserve the silk during long periods of time. All of them involve the application of aggressive steps, such as sun exposure, hot steam from boiling water or hot air, during hours or even days. None of the scientific articles related to silk fibroin biomaterials has previously taken into account this fact in its section of materials and methods. In this work, the consequences of the stifling treatments most commonly used by the silk producing countries and companies are explored in depth, using fibroin films as biomaterial model. The protein degradation (visualised by SDS-PAGE) was dramatically increased in all the fibroin dissolutions produced from stifled cocoons; heavy and light chains of fibroin were specially degraded, reducing their presence along the lanes of the gel compared to the negative control (untreated fresh cocoons). Structural changes are also described for annealed silk fibroin films. The β-sheet content, analysed by means of infrared spectroscopy, was significantly higher when stifling was performed at higher temperature (70 °C and 85 °C). It is also exposed the impact of the stifling on the mechanical properties of the materials. Tensile strength and strain at break values were detected as significantly lower when this procedure was carried out by means of dry heat (85 °C) and sun exposure. On the other hand, and contrary to expectations, the proliferation of fibroblasts growing on the materials was improved by all the different stifling methods, compared to negative control, being this improvement, especially accentuated, on the films produced with fibroin purified from cocoons treated with dry heat.

## Introduction

Silk fibroin (SF) biomaterials are attracting the attention of numerous researchers around the world, especially over the past two decades. The silk of *Bombyx mori* (silkworm) is composed of two proteins that present interesting biological properties. The silk filament is constituted by fibroin and sericin, the second one, is the water-soluble protein, with a globular structure that holds the filaments of fibroin together. Moreover, SF is composed of two protein components: a heavy chain (390 kDa) and a light chain (26 kDa) present in a ratio 1:1 and linked by disulphide bonds. At the same time, six of these structural blocks are connected together through their interaction with the glycoprotein P25, also named as fibrohexamerin (25 kDa)^1^. The heavy chain contains 12 domains of repeated amino acid motifs, which constitute the crystalline region of the fibre. These crystalline regions alternate with other non-repetitive primary sequences, and therefore, less organised. This molecular structure is what gives the material a unique combination of mechanical strength and elasticity.

It has been proven that SF, apart from its obvious textile application, present uses of great interest in the field of biomedicine and tissue engineering. The advantages offered by fibroin are numerous, in relation to the most commonly used materials. It is a protein-based biomaterial, highly biocompatible, with excellent mechanical properties, biodegradable and it can be processed in different configurations (dissolutions, hydrogels, films, sponges, particles and nanofibers)^2,3^ according to the needs of the clinical condition to be treated or the tissue to be repaired. SF is also bioactive by itself promoting wound healing^4,5^ and its surface has reactive chemical groups to which peptides and growth factors can be covalently bound.

Reproducibility in research constitutes a basic requirement in order to create useful knowledge for the rest of the scientific community, whose accumulation generates consistent and feasible social improvements, but sometimes basic aspects, that guarantee the consistency of results and the reproducibility of the experiments, are ignored, causing knowledge with important “gaps”. In this sense, there are thousands of research articles and reviews that have explored the biomedical applications of SF^6–18^, nevertheless, there is a lack of information on some elementary points of silk manufacturing and its effects on the materials resulting from its processing. Some research groups have investigated the effects of different silk processing steps on the materials produced, always starting from the sericin removal (also known as degumming). Thus, differences have been analysed and detected for diverse degumming protocols^19–23^, raw silk dissolution methodologies^20,21,24^ or different refrigeration and ageing times in fibroin dissolutions^25,26^. Other authors have even studied the structural and thermal properties of SF films obtained from different sources (cocoon and waste silk fibres) as raw materials^27^. However, to our knowledge, the effects of the different heat treatments applied to silk cocoons, before long term storage, have not been previously studied in the field of biomaterials. In this sense, it would also be interesting to study the properties of silk proteins in carbonized state, since this type of materials have been described in the scientific literature as extreme biomimetic both in the case of fibroin^28^ and spongin^29,30^.

Cocoon stifling is the treatment applied to the cocoons in order to kill the pupae, preventing the emergence of moths and allowing to preserve the silk for longer periods of time, but, even being a treatment, in general, quite aggressive for silk, none of the works on fibroin biomaterials refers to it on its section of materials and methods. This fact denotes, either a lack of information in this respect or an omission of it. It is therefore assumed, erroneously, that there is no influence between the treatment of stifling and the mechanical, chemical or biological behaviour of the biomaterials produced. This procedure can be carried out in different ways. Some silk factories prefer to perform cocoon stifling by exposure to the sun during periods of time ranging from 3 to 5 days, depending on the temperatures and irradiation of the geographical location at a given time. This procedure was reported by Nguku *et al*. (2009) as the most common method to preserve cocoons all over the world because of the lack of sufficient preservation methods by the silkworm rearers^31^ but involves potential losses due to rodents, insects, rains or microorganisms. Moreover, this method decreases the quality on the sericin layers, and, at the same time the quality of the silk fibres, with reeling losses of cocoons^32^.

Other methodologies carried out for the stifling of silk cocoons involve the use of steam produced in a container with boiling water and a subsequent drying phase at room temperature for several days, or the direct use of hot air drying. In this last case the pupae are killed and the cocoon dried simultaneously^33^ and this methodology can be implemented within a wide range of temperatures. Some works have explored the influence of this factor on parameters indicative of the textile quality of the obtained silk^34^, applying temperatures ranging from 55 °C to 85 °C for 7 hours^35^ or between 60 °C and 75 °C^32^, but there are only a few works in the scientific literature comparing the different stifling methods and its effects on the obtained silk, always related to textile applications^31,33^. Nevertheless, to our knowledge, the potential effects of these treatments on SF biomaterials used for tissue engineering or biomedical research have not been investigated yet.

In this work, the consequences of the different stifling treatments on the properties of SF films used as biomaterial model, are explored in depth, in terms of protein degradation, structural changes, mechanical properties and biocompatibility. It is intended, in this way, to delve into the factors that affect the reproducibility in the manufacture of SF biomaterials, making a call to scientific community about the importance of addressing these basic aspects of silk processing in order to obtain controlled and consistent results.

## Materials and Methods

### Cocoon stifling and silk fibroin processing

Cocoons of *B*. *mori*, were obtained from silkworms reared in the sericulture facilities of the IMIDA (Murcia, Spain) with mulberry leaves. The eggs (Italian poly-hybrid (79 × 719) × (126 × 125)) were kindly supplied by Dr. S. Cappellozza (CRA-API).

The obtained cocoons were stifled in different ways in order to analyse how this treatment affects the properties of the fibroin biomaterials made from them. A negative control of non-stifled fresh cocoons was used to compare with the other treatments. Batches of 5 g of cocoons were used for this purpose, removing the pupae prior to silk processing. This procedure was carried out following the three most commonly used stifling methodologies in sericulture: sun exposure, water vapour exposure and dry heat (at different temperatures), as explained below:

#### Sun exposure

The cocoons were exposed to the sun arranged on a thin layer for 5 days, at the beginning of August in Murcia (Spain), coinciding with the summer season and moving them daily to ensure homogeneous exposure to the sun. In this period of time the cumulative daily average solar radiation was 26.2 MJ/m^2^, the average value of maximum radiation was 908.6 w/m^2^ and the average daily solar radiation was 303.6 w/m^2^. The temperature ranged between 38 °C (maximum) and 24 °C (minimum), with an average value of 31 °C. The relative humidity values fluctuated from 58% to 20%, with an average value of 37%. These data were recorded from the MU62 meteorological station (Agrometeorological Network of IMIDA) located in the same place where the experiment was performed.

#### Water vapour exposure

This stifling method was carried out using a 1 L beaker containing boiling water. A metallic net was used to place the cocoons suspended on the released steam during 3 hours. The temperature reached 85 °C near the cocoons. Subsequently, they were allowed to dry at room temperature for 24 hours.

#### Dry heat exposure

Heat drying was performed using a mechanical convection oven (redLINE RF 115). The temperatures and duration of the treatments were chosen based on previous works applied to the textile field^32,35^. Cocoons were introduced in the oven at 55 °C, 70 °C or 85 °C for 7 hours. In order to evaluate to what extent the applied temperature affects the resulting biomaterial within the same type of stifling method (in this case by means of dry heat).

After the stifling treatment, cocoons were chopped into 4 pieces and boiled in 0.02 M Na_2_CO_3_ for 30 min, to remove the sericin. Then, the raw SF was rinsed thoroughly with water and dried at room temperature for 3 days. The extracted SF was dissolved in 9.3 M LiBr (Acros Organics) for 3 h at 60 °C, yielding a 20 wt. % dissolution that was dialysed against distilled water for 3 days (Snakeskin Dialysis Tubing 3.5 KDa MWCO, Thermo Scientific), with eight total water changes (at 4 °C). The resultant 6 wt. % SF dissolution was used for the preparation of the SF films and to perform the SDS-PAGE.

### Films preparation

SF films were obtained by casting 1.3 mL of 6 wt. % SF aqueous dissolution on a plastic Petri dish, 5.8 cm in diameter, to give a 25 µm thick film (once dried at room temperature)^36^. Then it was performed a water annealing step by placing the SF films in a water-filled desiccator in vacuum conditions for 24 h, in order to produce water insoluble materials. These were used to characterise the mechanical properties and the infrared spectra.

A second type of films were manufactured in order to develop the cell culture experiments. In this case 48-well culture plates were used as a template. 150 µL of 2 wt. % SF aqueous dissolution were added per well and after drying at room temperature they were water-annealed in the same way, previously described, giving rise to 25 µm thick films immobilized on the culture plate and ready to be sterilized and seeded with the cells.

### Sodium Dodecyl Sulfate PolyAcrylamide Gel Electrophoresis (SDS-PAGE)

The SF dissolutions made from cocoons with different treatments were used to analyse the protein degradation of purified SF by means of SDS-PAGE (see Supplementary Information, Fig. S1). It was performed, according to the Laemmli protocol^37^ with a 4–20% gradient acrylamide gel (Amersham GE-HC). The setup used was a horizontal Gel-Box electrophoresis chamber (Amersham GEHC). After electrophoresis, the gels were stained with 0.25 wt.% Coomassie Brilliant Blue (Acros Organics, Belgium). The molecular-weight marker used was the SpectraTM Multicolor Broad Range Protein Ladder (Thermo Scientific). Protein concentrations were unified at 30 µg per lane; these samples were loaded under denaturing conditions by adding β-mercaptoethanol (10 vol.%) to the loading buffer and heating at 95 °C for 5 min just before the electrophoresis. Pictures of the gels were processed with ImageJ software to determine the peptide sizes of regenerated SF dissolutions comparing them with the protein marker as previously reported by our research group^24^.

### Attenuated total reflectance Fourier transformed infrared spectroscopy (ATR-FTIR)

ATR-FTIR was used to analyse the potential structural differences of annealed SF-films attributed to the stifling treatments applied to the cocoons. Each spectrum was acquired on a Nicolet iS5 spectrometer, equipped with an iD5 ATR accessory (Thermo Scientific, USA) controlled with OMNIC software (Ver. 9.3.30), measuring in absorbance mode with a resolution of 4 cm^−1^, a spectral range of 4000–550 cm^−1^, and 64 scans. The analysis was finally focused in the amide I region (1720–1585 cm^−1^) and Fourier self-deconvolution (FSD) was automatically performed using Gaussian/Lorentzian line shape by means of the function “peak resolve”^25^. In order to measure the relative areas of the amide I components, FSD spectra were then curve fitted. The positions (in cm^−1^) of the band maxima in the deconvoluted spectra were made to correspond to the frequency of the minima in the second derivative of the undeconvoluted spectra. Finally, the deconvoluted amide I spectra were area normalized, and the relative areas of the single bands were used to calculate the fraction (%) of the secondary structural elements. Vibrational band assignments were based on the data summarized by Hu *et al*.^38^.

### Evaluation of mechanical properties of SF films

Tensile tests were performed using a universal test frame machine (Qtest; MTS Systems, Eden Prairie, MN, USA). The mechanical properties of specimens (10 mm × 30 mm) were recorded with a crosshead speed of 0.1 mm·s^−1^ and a load cell of 200 N, under ambient conditions. The thickness of each piece of film was determined with an electronic digital micrometer (Mitutoyo Digimatic Micrometer 0–25 mm, resolution of 0.001 mm and an accuracy of ±2 μm). The elastic modulus (GPa), tensile strength (MPa) and strain at break (%) were determined using the stress-strain curves. Elastic modulus was calculated in the linear elastic portion of the stress-strain curves generated. Each test was performed at least three times per condition.

### Cell culture

#### Routine culture of HDF cells

Human dermal fibroblasts (HDF (106-05a) cell line, ECACC N° 06090715) were chosen for the cell culture study. Viability and cell number were determined by trypan blue staining in a Neubauer chamber and the cells were tested for the absence of mycoplasma before performing the experiments. The HDF cells were seeded in 25 cm^2^ flasks at a density of 5∙10^3^ cells∙cm^−2^ in DMEM/F-12 (1:1) expansion medium (supplemented with 5% FBS, 100 U∙mL^−1^ penicillin and 100 μg∙mL^−1^ streptomycin) at 37 °C in a humidified atmosphere with 5% CO_2_. The medium was carefully replaced twice a week and cells were allowed to grow until the culture reached 80% confluence. All the chemicals used for cell culture were purchased from Sigma-Aldrich (St. Louis, MO, USA) and Gibco (Paisley, UK); Nunc (Roskilde, Denmark) provided the culture plates.

#### Biocompatibility and proliferation assays

SF films were sterilised by immersion in 70% (vol.) ethanol during 10 minutes and washed twice with sterile 1X PBS dissolution before the seeding. The cells were detached using 0.05% trypsin/EDTA and seeded on the films at a density of at 5∙10^3^ cells∙cm^−2^ with 1 mL of DMEM/F-12 (1:1) expansion medium. Tissue culture polystyrene substrates (TCPS) were also seeded to be used as positive controls. Cell proliferation was evaluated 2 d, 4 d, 7 d and 10 d after seeding, using PrestoBlue (PB) reagent (Invitrogen, Thermo Fisher Scientific,Waltham, MA, USA). This is resazurin-based membrane permeable solution which does not require cell lysis. PB quantitatively analyses proliferation of metabolically active cells by mitochondrial reduction of resazurin to a red fluorescent compound called resorufin. As a consequence, the reagent exhibits a change in colour, as well as a shift in its fluorescence. Following the manufacturer’s protocol, a 10% solution of PB was added to the wells and incubated for 4 hours at 37 °C in a 5% CO_2_ humidified atmosphere. The solution was then removed and relative fluorescence (RF) was measured using a Synergy MX microplate reader (Biotek Instruments, VT, USA) with an excitation wavelength of 570 nm and an emission wavelength of 610 nm.

#### Cellular morphology and adhesion

In an effort to visualise the appearance and adhesion of cells growing on the surface of SF films produced from different treatments, cells were fixed using 4% parafomaldehyde in 1X PBS, 7 days after cell seeding. Then, at least 3 films per treatment, were stained with Neurite Outgrowth Staining Kit according to the manufacturer’s instructions (Molecular probes, Carlsbad, CA, USA). The shape of the cells was monitored via bright orange staining of outer cell membrane surfaces and samples were imaged in a fluorescence microscope (Nikon Diaphot-TMD, Japan).

### Statistical analysis

For the statistical analyses, SPSS software was used. As the data followed the normality requirement (Kolmogorov-Smirnov), they were compared by means of the parametric test ANOVA followed by Bonferroni’s post hoc multiple t-test.

## Results and Discussion

### Macroscopic appearance throughout the silk processing

**Figure 1:**
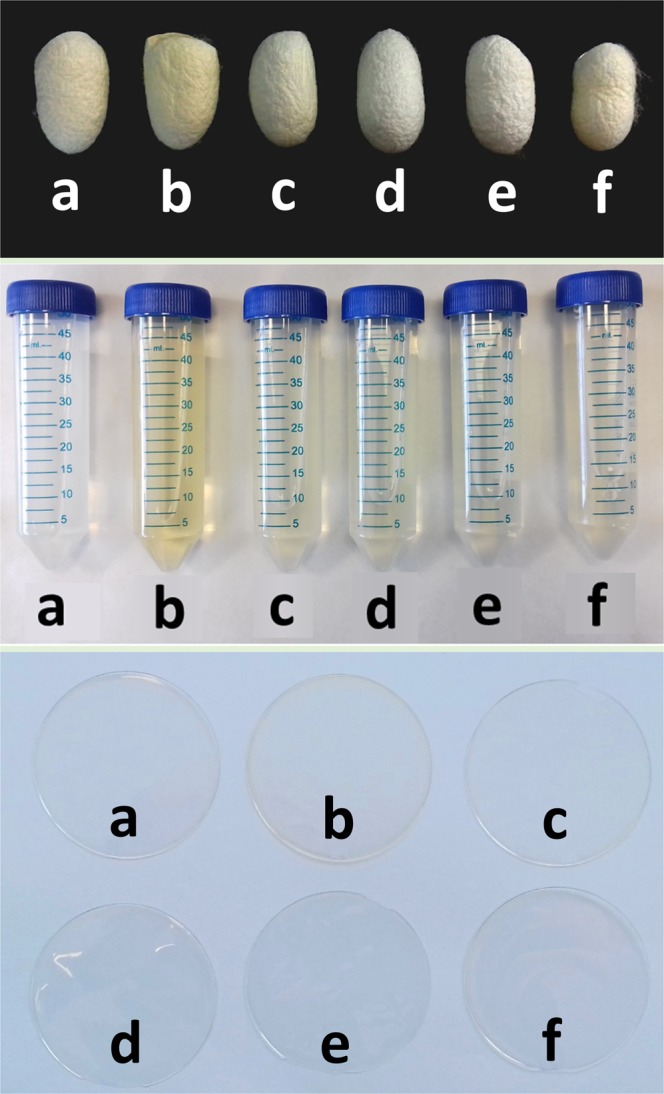
Illustrative images of silk cocoons stifled by means of different methodologies (upper picture), macroscopic views of fibroin dissolutions (middle picture) and films obtained from these cocoons (bottom picture). The different treatments consisted of non-stifled cocoons (**a**) and cocoons stifled by means of sun exposure (**b**), water vapour (**c**) or dry heat at 55 °C (**d**), 70 °C (**e**) or 85 °C (**f**).

The silk cocoons were stifled, as explained in the section of material and methods, in order to evaluate to what extent this handling can affect the properties of the biomaterials obtained from them. From the moment they were treated, there were clear differences in their coloration. Some of them changed, from displaying an intense white colour to becoming yellowish and with a lower brightness. This fact was especially accentuated in the cocoons treated by exposure to the sun and also, although to a lesser extent, in those treated with water vapour and with dry heat at 85 °C (Fig. 1). In fact, the dissolutions of aqueous SF obtained after the degumming of these cocoons also presented these yellowish tones, especially in those obtained after exposure to the sun. The films fabricated after this stifling treatment also showed the same macroscopic appearance. These point is in agreement with what has been described by other authors in the field of textile sericulture, who stated that ultraviolet rays can affect the colour and cleanness of silk^35,39^.

### Protein degradation (SDS-PAGE)

**Figure 2:**
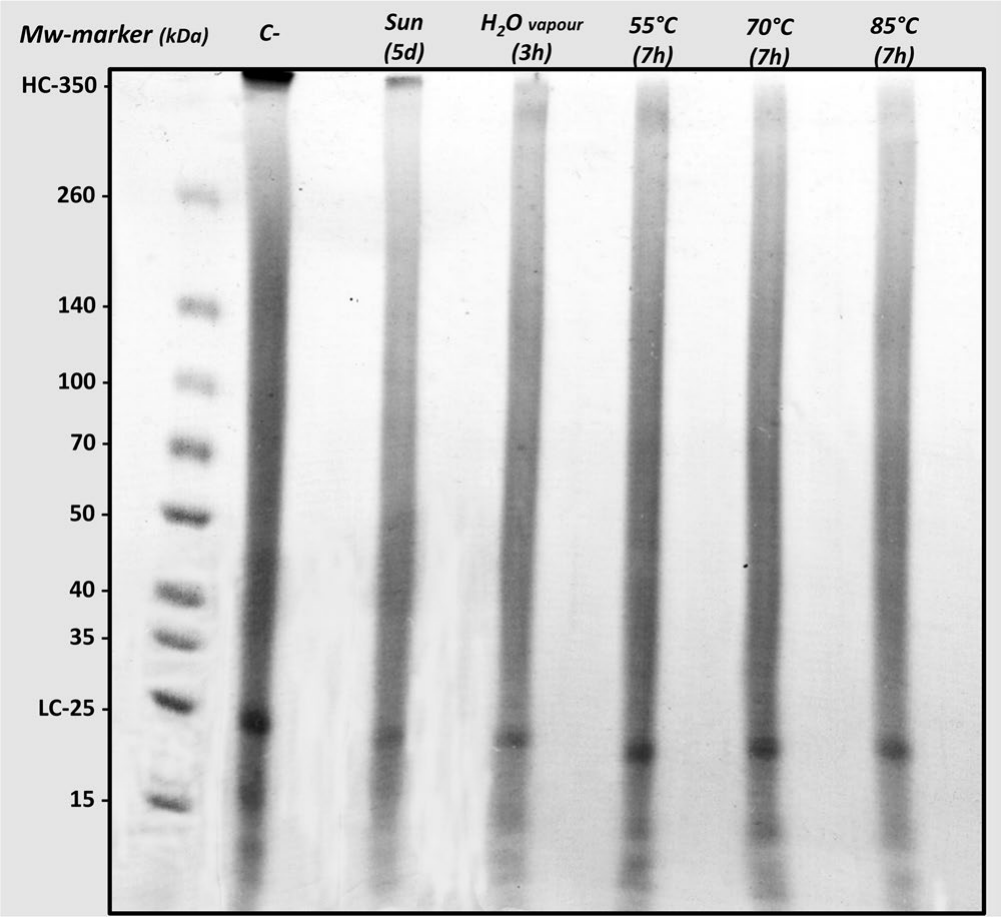
SDS-PAGE analysis of SF aqueous dissolutions obtained from cocoons stifled in different ways. “C-” refers to negative control obtained from non-stifled fresh cocoons.

SDS-PAGE is commonly employed to analyse the degradation of regenerated SF in the scientific bibliography. In previous studies, it has been shown, by means of this technique, that the degumming time dramatically increased the degradation of fibroin^20–22^, the way in which the different dissolution protocols influenced the integrity of the protein^20,24,40^ or in what manner the conservation time of SF aqueous dissolutions also inherently involved degradation processes^25^. Therefore, this methodology was applied in order to evaluate how cocoon stifling affects the integrity of the resulting fibroin after being dissolved and dialyzed. Figure 2, shows the appearance of the SDS-PAGE after staining it with Coomassie Brilliant Blue. Clear differences were observed in all the stifling treatments compared to fibroin obtained from fresh untreated cocoons (negative control).

The heavy and light chains underwent a considerable degradation, besides detecting variations in the peptide degradation profiles. In order to quantify these visually detected variations, an image analysis was carried out^24^, taking into account the optical densities of heavy and light chains (350 kDa and 25 kDa, respectively) and those of the peptide gradients between them and below 25 kDa. These data were quantified as percentages of optical density of each lane. The ratio light chain/heavy chain (LC/HC) was also calculated (Table 1). This calculation was carried out in an attempt to correlate the presence, to a greater or lesser extent, of the light chains in relation to the heavy chains, given that some studies have linked the viability and adhesion of cells growing on fibroin biomaterials with the greatest presence of light chains of fibroin^41^. This fact will be discussed in more detail below.

**Table 1:** Percentage of SF polypeptides, including values obtained for heavy chains and light chains, detected in SF aqueous dissolutions, depending on the treatment applied to the silkworm cocoons (before the degumming and dissolution of the raw SF).

	350 kDa *heavy chain (HC)*	(350-25) kDa	25 kDa *light chain (LC)*	<25 kDa	*LC/HC*
Negative control	4.22 ± 0.07	68.31 ± 1.24	10.22 ± 0.68	17.25 ± 0.57	2.42 ± 0.15
Sun exposure (5 d)	1.86 ± 0.20*	73.01 ± 0.82*	7.58 ± 1.07	17.55 ± 1.96	4.07 ± 0.20
Water vapour (3 h)	1.05 ± 0.21*	74.15 ± 1.06*	7.21 ± 0.84	17.57 ± 0.16	6.95 ± 0.68
Dry heat 55 °C (7 h)	0.50 ± 0.04*	74.67 ± 0.86*	7.39 ± 1.23	17.44 ± 0.58	14.68 ± 1.25*
Dry heat 70 °C (7 h)	0.32 ± 0.02*	74.48 ± 0.85*	6.47 ± 1.42*	18.73 ± 0.61	20.14 ± 3.40*
Dry heat 85 °C (7 h)	0.36 ± 0.08*	76.12 ± 0.67*	5.82 ± 0.61*	17.70 ± 0.03	16.25 ± 2.19*

The values obtained for the ratio between the percentages of light and heavy chains (LC/HC) are expressed in the last column.

*Statistically different values compared to negative control (Bonferroni, p < 0.01).

The heavy chains of fibroin were degraded in all treatments, compared to the negative control, and statistically significant differences were detected (Bonferroni, p < 0.01). This decrease in percentage of optical density of each lane of the gel was especially pronounced in the case of treatment with dry heat (70 °C and 85 °C), presenting values lower than 0.5%, which is a considerable decrease if we take into account that in the case of fibroin obtained from untreated cocoons its value was 4.2%. The peptides derived from the degradation of the heavy chains (350-25 kDa) increased significantly compared to the negative control in all the stifling treatments (Bonferroni, p < 0.01) reaching a maximum value of 76.1% in the case of cocoons stifled with dry heat at 85 °C. The light chains also suffered a considerable degradation with the different stifling treatments and a decrease in the average values of percentage of optical density was observed in all the studied methodologies, but in this case it was only detected as statistically significant in the treatments with dry heat at 70 °C and 85 °C comparing them with the negative control (Bonferroni, p < 0.01). On the contrary, no differences were detected in the percentage of fibroin peptides less than 25 kDa (Bonferroni, p > 0.01).

On the other hand, the LC/HC ratio values were detected as statistically different from the negative control (Bonferroni, p < 0.01) in the three treatments employing dry heat for the cocoon stifling (55 °C, 70 °C and 85 °C), thus detecting a greater presence of light chains of fibroin with respect to the heavy ones, in these SF dissolutions.

### Analysis of structural differences in SF films (FTIR-IR)

Infrared spectra of the annealed SF films, produced from cocoons stifled in different ways, were recorded in order to visualise the potential changes in terms of percentage of secondary structural elements. The analysis was focused in the amide I region (1720–1585 cm^−1^) and FSD was automatically performed based on the protocol described by Hu *et al*.^38^ (see Supplementary Information, Fig. S2), this is widely employed with the same purpose by other authors^25,42–44^, whose results are in the range of the ones obtained in this experiment. As can be observed in Table 2, the content of secondary structures was quite similar to the negative control in all the treatments except those involving dry heat at 70 °C and 85 °C. The β-sheet content was estimated around 30%, the random coil content ranged between 24% and 26% and the side chains represented 16–18% of the amide peak I. However, in the treatments involving cocoon stifling with dry heat at 70 °C and 85 °C the content in β-sheet was dramatically increased up to 40%, while the content in random coil decreased up to 20%, as well as the content in side chains (around 12%). These differences were statistically significant with respect to the negative control (Bonferroni, p < 0.01). Additionally, it should be noted that the content in α-helix (9–10%) and turns (17–18%) was statistically equal in all treatments (ANOVA, p > 0.01). This fact can be understood taking into account other works that have already described an increase in β-sheet conformation associated with the thermal degradation of the silk protein, behaviour that is often observed during the conformational transition of silk proteins from unstable random coil structures to a stable β-sheet crystal structure^28^. A similar condition has been described by other research groups working with elastin-like-peptides (ELP), stating thermal structural transitions towards an increase in β-sheet content related to the increase in the temperature during the incubation of ELP dissolutions^45^.

**Table 2:** Relative content of secondary structures of the annealed films made with SF dissolutions obtained from cocoons stifled with different methodologies.

	β-sheet (%)	Random coil (%)	α-helices (%)	Turns(%)	Side chains (%)
Negative control	30.9 ± 0.6	24.4 ± 0.4	9.3 ± 0.4	17.6 ± 0.4	17.9 ± 1.0
Sun exposure (5 d)	32.3 ± 0.7	23.7 ± 0.4	9.5 ± 0.4	18.2 ± 0.7	16.3 ± 0.6
Water vapour (3 h)	31.6 ± 1.8	25.1 ± 2.1	9.3 ± 0.9	17.3 ± 0.2	16.7 ± 0.5
Dry heat 55 °C (7 h)	30.4 ± 0.4	26.6 ± 0.3	8.6 ± 0.1	17.3 ± 0.2	17.1 ± 0.4
Dry heat 70 °C (7 h)	40.3 ± 0.1*	20.1 ± 0.2*	10.5 ± 0.1	16.7 ± 0.1	12.5 ± 0.1*
Dry heat 85 °C (7 h)	40.4 ± 0.3*	20.6 ± 0.8*	10.0 ± 0.8	16.7 ± 0.4	12.3 ± 0.3*

Data are obtained from Fourier self-deconvolution of the infrared spectra covering the amide I region. Values are expressed as mean ± standard deviation (n = 3).

*Statistically different values compared to negative control (Bonferroni, p < 0.01).

This phenomenon of increase in the crystalline fraction of fibroin has been previously identified by other authors who detected a transformation from random coil to β-strand along the time of conservation of SF dissolutions^26^, as well as by our research group in a related work, using electrospun mats as a model material^25^. Moreover, it could be hypothesized that the increase in the β-sheet content may also be caused by the thermal degradation processes to which fibroin is subjected during cocoon stifling, especially affecting the amorphous fraction of the protein and thus enriching the β-sheet content whose molecular structure is much more stable and difficult to degrade.

### Mechanical properties of SF films

**Figure 3:**
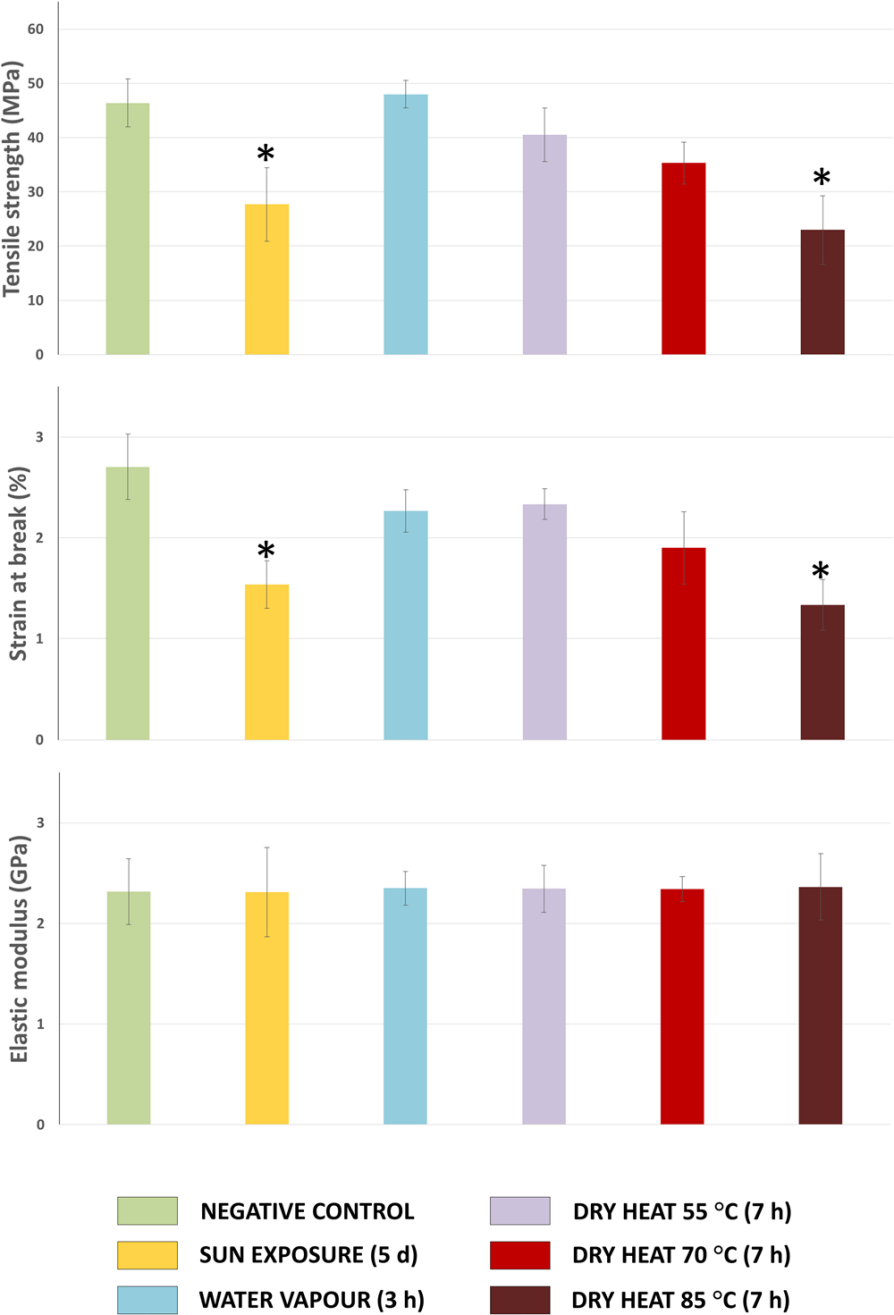
Image illustrating the results regarding the mechanical properties of the annealed films obtained using fibroin from cocoons stifled in different ways. *Statistically different values compared to negative control (Bonferroni, p < 0.01).

Tensile tests were performed to evaluate the mechanical properties of the SF films produced from the fibroin dissolutions of differentially treated silk cocoons. It was intended to correlate in this way, the phenomena of fibroin degradation associated with cocoon stifling, as well as the content of secondary structures (analysed by FTIR), with the mechanical behaviour of the materials produced, in order to clarify to what extent the stifling can affect to this aspect. Table S1 (see Supplementary Information) summarizes the results obtained after the analysis of stress-strain curves generated, and the differences detected are clearly illustrated in the Fig. 3. First, it should be mentioned that the results obtained for tensile strength, strain at break and elastic modulus are in the range of those previously described by other authors for this kind of materials^23,36,46,47^.

The values of elastic modulus ranged from 2.3 to 2.5 GPa and were statistically equivalents in all the studied cases (ANOVA, p > 0.01). On the other hand, the values of strain at break, or what is the same, elongation at break, oscillated between the maximum value, around 3%, calculated for the films obtained from the fresh cocoons (negative control) up to the minimum value of 1% recorded for films made with fibroin from cocoons heat-treated at 85 °C for 7 h. The decrease in the average value of strain at break, detected in all the films produced from stifled silk cocoons, denotes the importance of the protein degradation on this parameter^23^. Statistically significant differences were detected, in the comparison with the negative control, in the cases of cocoon stifling performed by means of sun exposure (1.5%) or application of dry heat at 85 °C (Bonferroni, p < 0.01), being much lower the values of strain at break reached in these circumstances (Fig. 3).

The average value obtained in the case of the treatment at 70 °C was also especially low (1.9%). This fact is totally correlated with the results obtained after the FTIR analysis, whose lower values in random coil content were detected in these same treatments previously mentioned. This datum makes sense given that the non-crystalline structures of fibroin are largely responsible for the elasticity of the materials produced from it. This affirmation is also supported by Koh *et al*. who stated that the extensibility and toughness of silk materials are governed mainly by the semi-amorphous matrix of fibroin^47^.

Regarding the ultimate strength values, it is important to note that the trend was the same as that observed with the values of strain at break (Fig. 3). Reaching the lowest values, significantly different (Bonferroni, p < 0.01) to the negative control (46 MPa), in the case of cocoon stifling by means of exposure to the sun (28 MPa) and application of dry heat at 85 °C (23 MPa), being also especially low the average value obtained in the treatment at 70 °C (35 MPa), although not statistically different.

### Cell culture

A primary study of biocompatibility of SF films was performed using the HDF (106-05a) cell line seeded onto thin SF films obtained using fibroin aqueous dissolutions from silk cocoons stifled in different ways (as described previously). Those films were fabricated onto polystyrene culture plates and the proliferation of HDF cells growing on their surface was analysed by means of PB assay.

**Figure 4:**
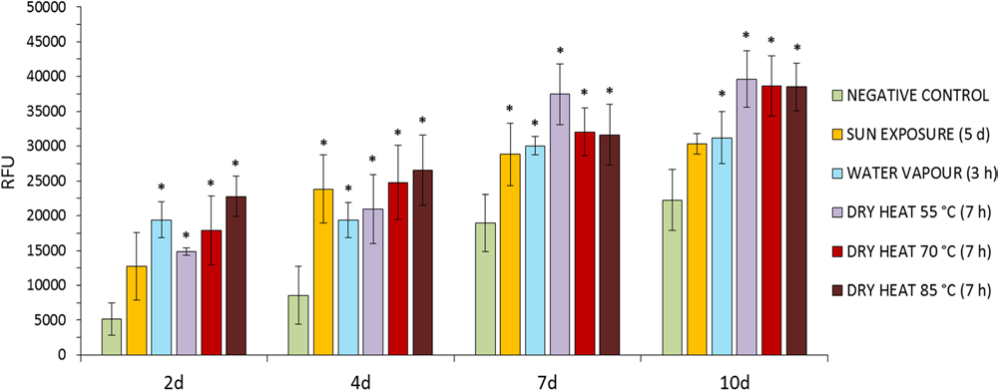
Proliferation of HDF cell line growing onto SF films obtained from cocoons stifled by means of different treatments. The study was performed at 2, 4, 7 and 10 days after seeding. Data are expressed as the average values of relative fluorescence units (RFU 570–610 nm) ± SD (n = 5) of the PrestoBlue (PB) test. *Statistically different values respect to cells growing on negative control (films obtained from fresh cocoons) at each experimental time (Bonferroni, p < 0.05).

Figure 4 presents the results of the proliferation test at 2 days, 4 days, 7 days and 10 days after cell seeding on the SF films. In an early phase of the proliferation experiment (2 days after cell seeding), it was detected a tendency towards higher relative fluorescence units in the case of cells growing on films made with fibroin from stifled cocoons, which corresponds to a greater cellular proliferation on these materials. The differences were statistically significant with respect to the negative control in the case of cocoons stifled with steam and with dry heat (Bonferroni, p < 0.05), the increase in cell proliferation was correlated with the increase in the temperature of stifling in this type of treatment as can be observed in Fig. 4. This fact is probably due to the greater degradation of fibroin in these cases, exposing a larger quantity of bioactive peptides^5^ related to improvements in the initial adhesion on the biomaterials and stimulating cell proliferation.

During intermediate periods of the proliferation study (4 d and 7 d), the tendency described above was confirmed, with better cellular proliferation observed in the films produced from stifled cocoons. Statistically significant differences (Bonferroni, p < 0.05) were detected in all cases, comparing the fluorescence values with those recorded for cells growing on the negative control films (produced from non-stifled fresh cocoons).

Finally, 10 days after the seeding, these differences in the cell proliferation on the films remained being statistically significant in the case of treatments with water vapour and dry heat, compared to the negative control (Bonferroni, p < 0.05). In addition to this fact, treatments that applied dry heat for the stifling (55 °C, 70 °C and 85 °C) showed fluorescence values also statistically different (Bonferroni, p < 0.05) and higher than those of the treatment with sun exposure (one of the most widely used treatments for cocoon stifling). It should be mentioned that at the end of the experiment (10 d) three clear trends were observed regarding the levels of cell proliferation. Firstly, and obtaining the best results from the study, there were the treatments with dry heat (55 °C, 70 °C and 85 °C). These were statistically equal between them (Bonferroni, p > 0.01), probably due to the fact that high levels of cell confluence were reached, thus homogenizing the differences observed among them in previous phases of the experiment. Secondly, films obtained from cocoons treated by exposure to water vapour and sun exposure were statistically equivalent at this point of the study (Bonferroni, p > 0.01) and they also improved the proliferation levels of those obtained from fresh cocoons (negative control).

**Figure 5:**
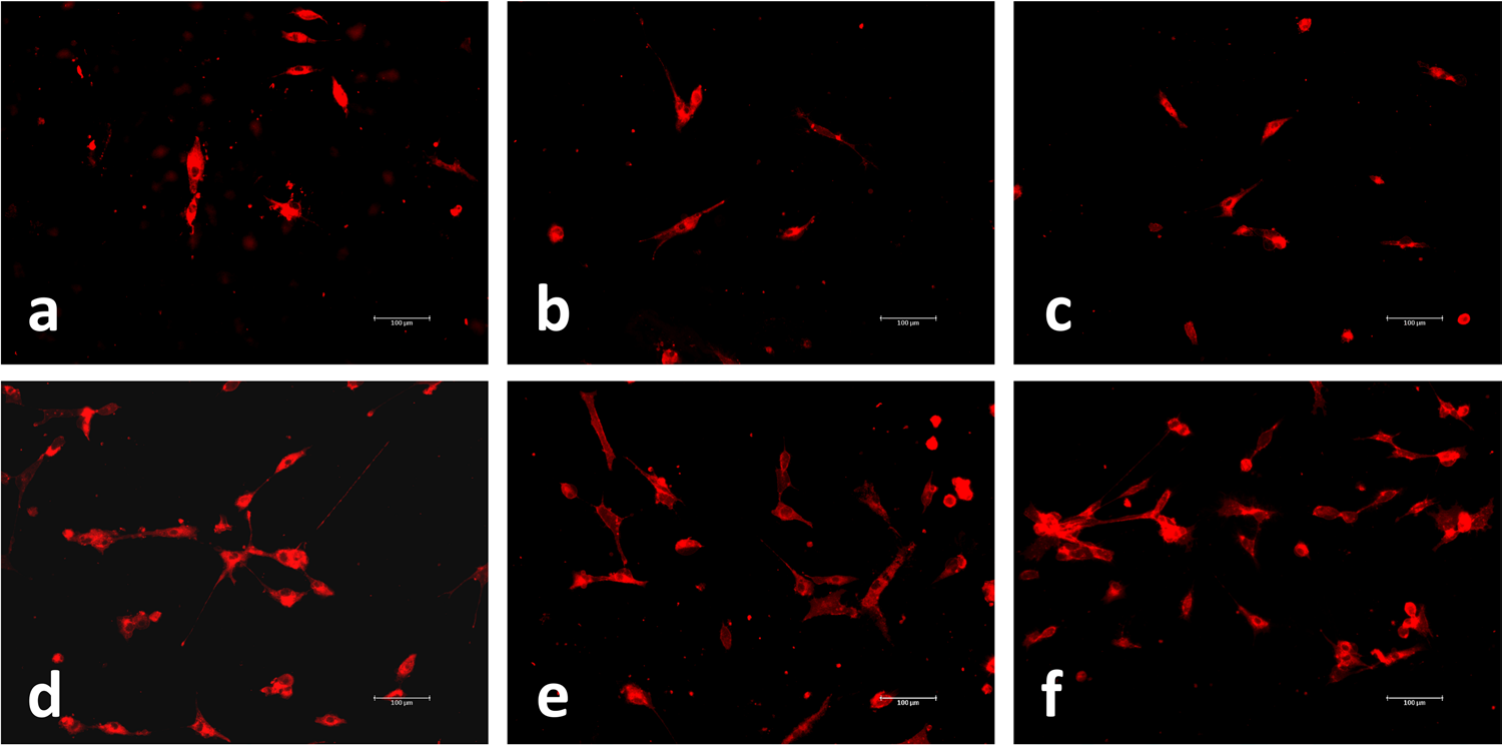
Micrographs of HDF cells growing on fibroin films produced from non-stifled cocoons (**a**), cocoons stifled by means of sun exposure (**b**), water vapour (**c**) or dry heat at 55 °C (**d**), 70 °C (**e**) or 85 °C (**f**). Pictures were acquired 7 days after the seeding (10x).

In order to confirm the results obtained in the PB assay, fluorescence micrographs of the cells in culture were acquired 7 days after the seeding (Fig. 5). As expected, a smaller number of cells was observed on the negative control films, with fewer connections between them and less expanded membranes. In the case of films obtained from cocoons stifled by means of water vapour and sun exposure, cell confluence levels were slightly higher than those obtained in the negative control and the main difference was the observation of a better cellular expansion and adhesion. Finally, in the cases of cells growing on films obtained from cocoons stifled by means of dry heat (at 55 °C, 70 °C and 85 °C) a much higher cell proliferation was observed, with highly expanded cells and emitting filopodia with multiple connections with nearby cells.

The explanation underlying the improvements in proliferation observed on some films made with stifled cocoons and the suboptimal behaviour of cells growing on those obtained from fresh cocoons can be found in the greater or lesser presence of heavy and light chains of fibroin in their composition. Wabdua *et al*.^41^ stated that fibroin scaffolds fabricated only with heavy chains had lower cell adhesion and the cells exposed to culture medium containing extracts of these scaffolds presented viability values slightly reduced when compared with scaffolds and extracts produced with only light chains or mixtures of both. In this sense, it is observed in Table 1 how the stifling treatments greatly degrade the heavy chains of fibroin, being inversely correlated the presence of the same in the materials with the levels of proliferation described. Therefore, the smaller is the amount of intact fibroin heavy chain present in the composition of the film, the greater is the cellular proliferation detected. Hence, cocoon stifling treatments improved the biocompatibility of the materials produced, to the detriment of certain mechanical properties. That is why this work presents a simple calculation in order to understand the biocompatibility results obtained according to the relationship between the presence of light and heavy chains in the composition of SF films (LC/HC ratio). Observing the values of LC/HC ratio presented in Table 1, an approximate positive correlation can be established with the cell proliferation levels obtained in each treatment. The highest values of LC/HC ratio are coincident with the highest levels of cell proliferation, obtained in the treatments with dry heat at 55 °C, 70 °C and 85 °C (14.68, 20.14 and 16.25, respectively). Values that are statistically superior to those of the negative control (Bonferroni, p < 0.01). On the other hand, the values obtained for the treatments with water vapour (6.95) and sun exposure (4.07) were slightly higher than the negative control (2.42), but not statistically different, being this increase enough to induce a significant improvement in the biocompatibility and cell proliferation of the materials produced from these silk cocoons.

Therefore, it could be concluded that cocoon stifling treatments employing dry heat promote improvements in the biocompatibility of the materials manufactured from them, given the marked degradation of the heavy chains of silk fibroin, whose presence in the fibroin biomaterials is associated with a reduction in the biocompatibility of the same^41^, as well as the presence of more degradation peptides of fibroin, widely described in the scientific literature as enhancers of cell proliferation^5,48^.

## Conclusions

In this work, we have explored in depth the consequences of the application of the most commonly used methods for cocoon stifling in the biomaterials of fibroin produced from these silk cocoons. The protein degradation was dramatically increased in all the fibroin dissolutions produced from stifled cocoons; heavy chains of fibroin were specially degraded, as well as light chains, although to a lesser extent. This fact generates a series of consequences not only in the secondary structures of fibroin, but also in the mechanical properties and ultimately in the biocompatibility of the materials produced. The β-sheet content was significantly higher when stifling was performed by means of dry heat at higher temperatures (70 °C and 85 °C), with a significant reduction in the content of random coil and side chains. Tensile strength and strain at break were detected as significantly lower when this procedure was carried out by means of dry heat (85 °C) and sun exposure. On the other hand, and contrary to expectations, the proliferation of fibroblasts growing on the materials was improved by all the different stifling methods, compared to negative control (non-stifled fresh cocoons). This improvement was especially accentuated on the films produced with fibroin purified from cocoons treated with dry heat, and is correlated with the higher degradation of the heavy chains and the greater prevalence of light chains and degradation peptides in their composition.

Given the results obtained, it is intended to draw the attention of the research groups working on SF biomaterials to take into account the methodology of cocoon stifling to be applied or described in their protocols, since this step has been proven to be crucial in the reproducibility and achievement of the desired objectives in tissue engineering research with fibroin.

## Supplementary information

Supplementary information

## Data Availability

All data generated or analysed during this study are included in this published article (and its Supplementary Information files).
