# Supplementary material for: Effect of different cocoon stifling methods on the properties of silk fibroin biomaterials
Source: Sci Rep. 2019 Apr 30;9:6703. doi: 10.1038/s41598-019-43134-5 (PMC6491555; doi:10.1038/s41598-019-43134-5)
Supplement: Supplementary file 1 — Supplementary information [file 41598_2019_43134_MOESM1_ESM.pdf]

Effect of different cocoon stifling methods on the properties of silk fibroin biomaterials.

Salvador D. Aznar-Cervantes*, Ana Pagan, Beatriz Monteagudo Santesteban, José L. Cenis.

Instituto Murciano de Investigación y Desarrollo Agrario y Alimentario (IMIDA), Department of Biotechnology. La Alberca (Murcia), E-30150, Spain.

**Corresponding Author: Salvador D. Aznar-Cervantes. Tel: +34 968368568, e-mail: sdac1@um.es*

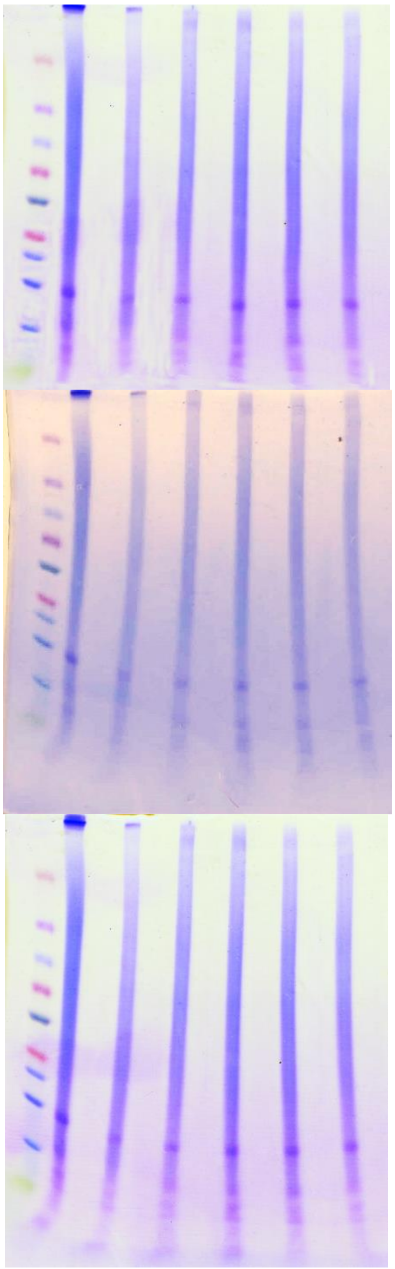

Figure S1. Images of full-length gels (SDS-PAGE) obtained for fibroin dissolutions from cocoons stifled by means of different protocols. The lanes observed from left to right correspond to: molecular weight marker, negative control, stifling by means of sun exposure, water vapour exposure and dry heat at 55ºC, 70ºC and 85ºC, respectively.

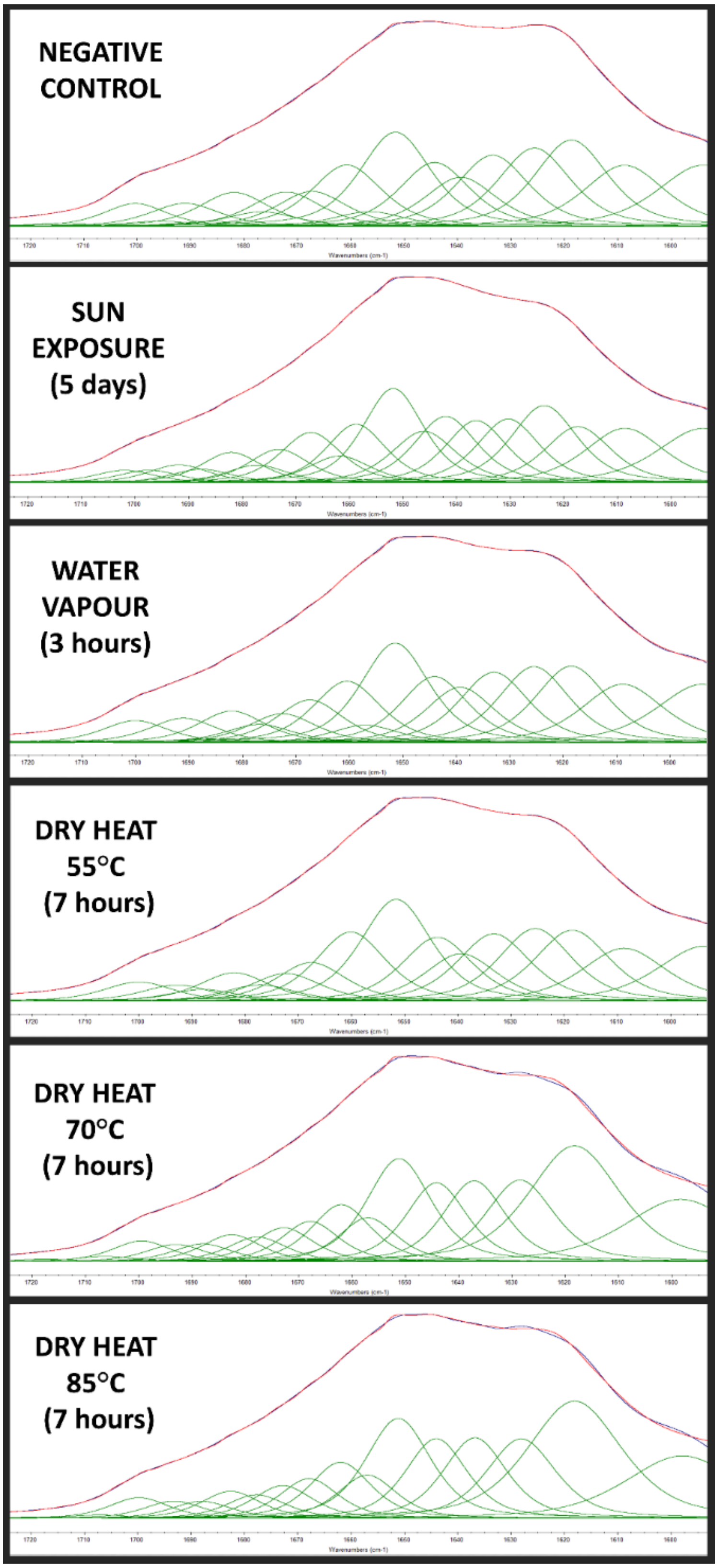

Figure S2. Illustrative images of Fourier self-deconvolution (amide I band of fibroin) performed to the annealed films produced with SF dissolutions obtained from cocoons stifled by means of different treatments.

Table S1. Mechanical properties of the annealed films made with SF dissolutions obtained from cocoons stifled with different methodologies. Data are expressed as average values ± standard deviation (n=3).

*statistically different values with the negative control (Bonferroni, p<0.01)

	**Tensile strength (MPa)**	**Strain at break (%)**	**Elastic modulus (GPa)**
**NEGATIVE CONTROL**	46.39 ± 4.40	2.70 ± 0.33	2.32 ± 0.33
**SUN EXPOSURE (5 d)**	27.70 ± 6.77*	1.54 ± 0.24*	2.31 ± 0.44
**WATER VAPOUR (3 h)**	47.97 ± 2.55	2.27 ± 0.21	2.35 ± 0.17
**DRY HEAT 55 °C (7 h)**	40.50 ± 4.96	2.33 ± 0.15	2.35 ± 0.23
**DRY HEAT 70 °C (7 h)**	35.30 ± 3.87	1.90 ± 0.36	2.34 ± 0.12
**DRY HEAT 85 °C (7 h)**	22.96 ± 6.28*	1.33 ± 0.25*	2.36 ± 0.33
